# Dietary supplementation with microencapsulated organic acids and essential oils improves sow productivity and nursery pig performance under commercial field conditions

**DOI:** 10.1186/s40813-026-00491-8

**Published:** 2026-02-12

**Authors:** Aprilia Rizky Riadini, Pornchalit Assavacheep, Kris Angkanaporn, Sukuma Samngamnim, Akaradet Seemacharoensri, Glenmer Bathan Tactacan, Ludovic Lahaye, Anongnart Assavacheep

**Affiliations:** 1 Department of Animal Husbandry, Faculty of Veterinary Science, Chulalongkorn University, Bangkok, 10330 Thailand; 2 Department of Veterinary Medicine, Faculty of Veterinary Science, Chulalongkorn University, Bangkok, 10330 Thailand; 3 Department of Veterinary Physiology, Faculty of Veterinary Science, Chulalongkorn University, Bangkok, 10330 Thailand; 4 Jefo Nutrition Inc., Saint-Hyacinthe, Canada

**Keywords:** Essential oil, Fecal bacteria population, Growth performance, Nursery pig, Organic acid, Sows

## Abstract

**Background:**

Organic acids (OA) and essential oils (EO) are used in swine diets to reduce diarrhea and support gut health. Although their benefits on growth and fecal microflora have been reported, data under commercial conditions especially involving both sows and nursery pigs remain limited. In this study, two experiments were conducted to investigate the effect of microencapsulated organic acids and essential oils P(OA + EO) on sow productivity and nursery pig performance. The first experiment, 334 sows were divided into 2 groups: (1) Control group, (2) Treatment group fed diet supplemented with P(OA + EO) from 7 days before to 28 days after farrowing. The second experiment, 3065 nursery piglets were divided into 2 groups: (1) Control group allocated into phase I (28–49 days of age) and phase II (50–70 days of age), (2) Treatment group fed the same phase I diet supplemented with 2 kg/ton P(OA + EO), and phase II with 1 kg/ton P(OA + EO).

**Results:**

Sows fed P(OA + EO) had significantly lower BCS and body fat losses compared with Control group. Percentage of sows with shoulder ulcer was lower in P(OA + EO) group. ADFI of sows in P(OA + EO) group was statistically higher than Control group. Number of pigs with diarrhea/litter, pre-weaning mortality, average weaned pigs/litter, average weaning weight/litter and average daily litter weight gain were all significantly improved by P(OA + EO). In the nursery pig experiment, ADFI and ADG were significantly higher, while FCR was significantly improved in P(OA + EO) group. At 28 days of age, total fecal bacterial population in P(OA + EO) group was significantly lower than Control group, whereas total fecal bacterial population, coliform, *E. coli* and *Lactobacillus spp.* were higher at 70 days. However, *Lactobacillus spp.*/Total bacteria ratio (L/T) and *Lactobacillus spp*./Coliform ratio (L/C) were similar between treatment groups at 28, 49, and 70 days.

**Conclusion:**

Supplementation of P(OA + EO) during lactation yielded significant improvements in sow productivity and suckling performance of piglets. Extending supplementation of P(OA + EO) into nursery period revealed positive impacts on growth performance of weaned piglets. These improvements could be attributed to the positive effect of P(OA + EO) on feed intake, leading to increased nutrient assimilation.

## Background

Antibiotics have been extensively employed in swine production for treatment, prevention, and control of diseases. Concerns arise about the potential for bacteria to acquire drug-resistant genes, which may have implications for human health through meat consumption or environmental exposure [1]. European countries have prohibited the use of antibiotics as growth promoters in livestock since 2006, and the US FDA has also imposed restrictions on antibiotic use in animal feed [2]. The limitations on antibiotic use undoubtedly impact the health and production of pigs.

Throughout the pig production cycle, a decrease in feed intake in productive sows due to farrowing stress not only affects colostrum production but also diminishes sow body fat [3]. Reduction in sow colostrum production leads to lower body weight, poor health, and subsequently, a higher mortality rate among suckling pigs. In addition to stress from sows, the weaning stage of piglets is critical for their survival due to the abrupt change in feed and the environmental shift, potentially resulting in post-weaning stress and diarrhea [4]. Numerous research studies have been addressed on ways to reduce diarrhea in weaned pigs by modifying intestinal microbiota to decrease the numbers of pathogenic bacteria such as *Escherichia coli* or Salmonella spp., while increasing the population of beneficial bacteria like *Lactobacilli* [5, 6]. Balancing the microbial population in the gastrointestinal tract (GI) positively affects growth performance by improving nutrient digestibility and reducing the incidence of diarrhea. Lower diarrhea during the weaning stage contribute to higher survival rates during the growing-to-finishing stage.

Blends of organic acid (OA) and essential oil (EO) in swine feed have been purposely used as additives to reduce diarrhea. Their ability to reduce stomach pH, or control pathogenic bacteria by suppressing nutrient transport and enzyme production through bacterial cell wall degradation [5], and aromatic properties to attract pig voluntary feed intake are promising future to commercial pig production industries [7, 8]. Using the latest technology, microencapsulated form of OA and EO deliberately delivers these active compounds to targeted sites in the gastrointestinal tract [9]. The lipid coating serves as protection matrix and delays OA and EO release along the different sections of the GI [10]. However, though several research had been attempted to prove that OA and EO blend could improve growth performance and fecal microflora in pigs, there are still limited numbers of research conducted in both sows and nursery pigs under commercial field conditions. This research was aimed to investigate the effect of dietary microencapsulated OA and EO on sow productivity and nursery pig performance.

## Materials and methods

### Animal ethics and protocol

Prior to the beginning of the field investigation, animal use (experiments 1 and 2) and protocols were reviewed and approved by Institutional Animal Care and Use Committee (IACUC no. 2031084) and Institutional Biosafety Committee (IBC no. 2031052), Faculty of Veterinary Science, Chulalongkorn University, Thailand.

### Experiment 1: Effect of P(OA + EO) on sow performance

#### Experimental animals and management

A total of three hundred and thirty-four healthy farrowing sows (Large white x Landrace) raised in a 6500 commercial farrowing-to-finish farm in Nakhon Nayok Province, Thailand, were used as experimental animals. Experimental sows were randomly divided into two groups; 1) Control group fed a standard corn–soybean meal based basal diet (*n* = 167), and 2). Treatment group fed the same Control diet supplemented on top with P(OA + EO) at a rate of 10 g/sow/day from 7 days before farrowing until 28 days after farrowing (*n* = 167). P(OA + EO) supplementation was provided to the treatment sows during the morning feeding. The sows were kept in an individual pen in a closed house with evaporative cooling system with separated water nipple and manual feeder. Farrowing pen was made of metal bar and T-bar slate flooring. All animals were raised in the same environmental and management conditions.

#### Experimental design

##### Feed and feedings

The basal diet was produced in the farm feed mill following GMP standard procedure in mash form. The feed was prepared every 2 days and delivered to the pig farm on production day. Feed and water were provided *ad libitum*. The P(OA + EO) is a commercially registered product of Jefo Nutrition Inc., Saint-Hyacinthe, Quebec, Canada under the brand name Porcinat+. P(OA + EO) is a microencapsulated blend of organic acids composed of citric acid, fumaric acid, malic acid, sorbic acid, and essential oils composed of eugenol, thymol and vanillin.

##### Sample collection and analysis

Feed samples were collected for proximate (dry matter, crude protein, crude fiber, crude fat and ash) and mineral (calcium and phosphorus) analyses at the beginning of experiment to determine nutrient composition (AOAC, 2016). Dry matter and ash were measured using an oven and a furnace, respectively. Crude protein was determined using Kjeldahl method. Crude fiber was analyzed using a raw fiber extractor. Calcium and phosphorus were analyzed by spectrophotometry.

**Fig. 1:**
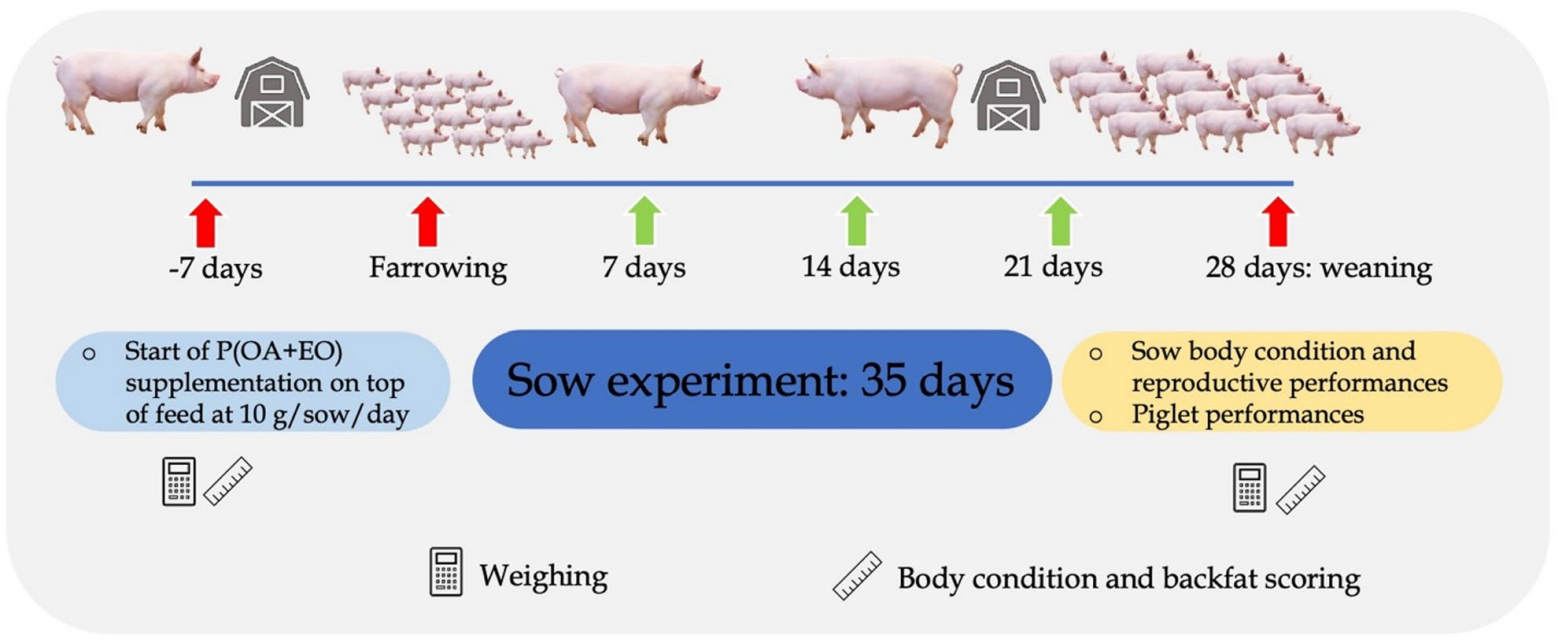
Summary of sow experiment design

The sow body condition was determined by measuring backfat (BF) thickness, body condition score (BCS), and shoulder ulcer. BF thickness was determined by using an ultrasound probe (Renco Corporation^®^, MN. USA) at the P2 position (6–8 cm away from body midline at the last rib curve) [11]. BCS was determined by scoring sow body size from 1 to 5, where a score of 1 indicated that the sow was emaciated and score of 5 indicated the sow was overly fat [12]. BCS and BF were recorded at the beginning (7 days before farrowing) and at the last day of the experiment (at weaning). Shoulder ulcer was evaluated with scores of 0 to 4, where a score of 0 indicated no lesion or scarring of skin over the tuber of the scapula, a score of 1 indicated no current lesion but previous scarring of skin over the tuber of the scapula, a score of 2 indicated the skin is reddened over the tuber of the scapula, a score of 3 indicated broken skin over the tuber of the scapula < 2.3 cm in diameter, and a score of 4 indicated broken skin over the tuber of the scapula > 2.3 cm in diameter [13]. Litter born alive was recorded at farrowing day. The numbers and weight of weaned piglets were recorded at day 28 of lactation (weaning). The experimental design is shown in Fig. 1.

The sow reproductive performance was measured by determining the total number of pigs born, number of born alive, percent stillborn, and weaning-to-service interval. Piglet performance data were also determined including average birth weight, percent of pig diarrhea, preweaning mortality, number of weaned pigs per litter, weaned pig weight, and average daily litter weight gain (ADLWG).

### Experiment 2: Effect of P(OA + EO) on performance of nursery piglets

#### Experimental animals and management

A total of three thousand and sixty-five healthy weaned piglets (Large white x Duroc x Landrace) were used. All animals were divided into two groups: (1) Control group fed a corn-soybean meal based basal diet allocated into phase I (28–49 days of age) and phase II (50–70 days of age) (*n* = 1539), and (2) Treatment group fed the same phase I diet supplemented with 2 kg/ton P(OA + EO), and phase II diet supplemented with 1 kg/ton P(OA + EO) (*n* = 1526). Each treatment had four replicates (with 380–385 pigs/ each replicate). All piglets were not derived from the sows used in experiment 1. Weaning piglets were randomly allotted in the pen with solid cement base with water bowl and feeder. Feed and water were provided *ad libitum*. All experimental animals were allocated in a completely randomized design.

#### Experimental design

##### Feed and feedings

The basal and the P(OA + EO) treated diets were produced in a farm feed mill following GMP standard procedure in mash form. The feed was prepared every day and delivered to the pig farm on production day. Feed and water were provided *ad libitum.*

#### Feed and fecal collection

##### Feed collection

Weaned pig feed samples were collected for proximate analysis [12] at the beginning of experiment to determine the nutrient composition of the experimental diets. The weights of given and refused feed were recorded.

##### Feed analysis

Feed samples were collected once for proximate (dry matter, crude protein, crude fiber, crude fat, and ash) and mineral (calcium and phosphorus) analyses at the beginning of experiment to determine nutrient composition [14]. Dry matter and ash were measured using an oven and a furnace, respectively. Crude protein was determined using Kjeldahl method Crude fiber was analyzed using a raw fiber extractor. Calcium and phosphorus were analyzed by spectrophotometry.

##### Fecal collection

**Fig. 2:**
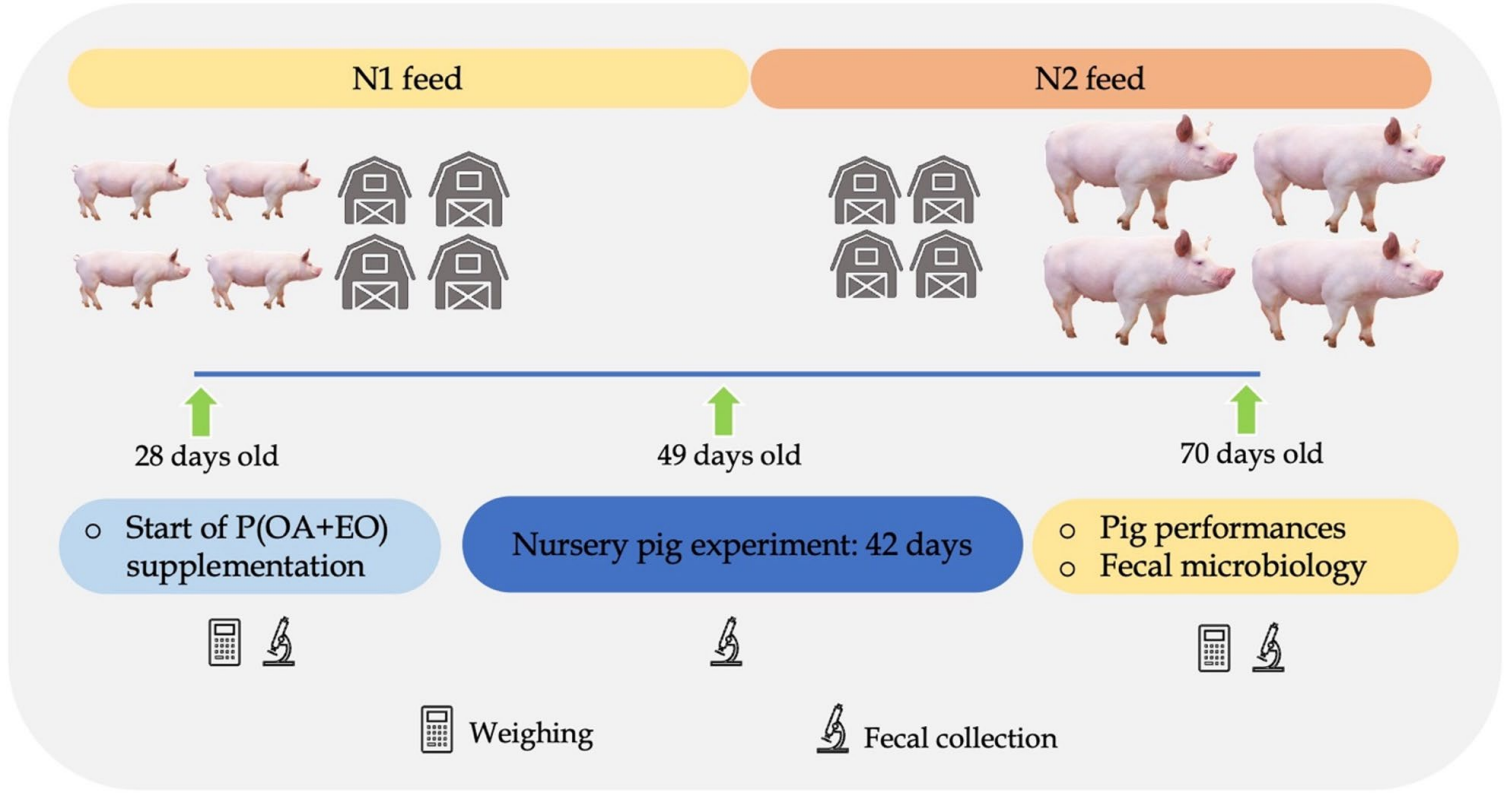
Summary of nursery pig experiment design

One hundred and eighty fecal samples were collected at 3 specific timepoints: at 28 days (beginning), 49 days (middle), and 70 days (the end of experiment). In each of the 4 replications, 15 samples from each treatment group were collected. Approximately, 1 to 2 g of feces were taken from the rectum and then transferred into plastic bags to determine number of total bacteria, coliform bacteria and *E. coli*, and Lactobacillus spp. The experimental design is shown in Fig. 2.

#### Fecal bacterial assessment

##### Total plate count (TPC)

One gram of sample was weighed, mixed, and centrifuged until homogenized with PBS and serially diluted 10-fold to obtain 10^− 4^ to 10^− 8^ dilution. One mL of sample suspension was transferred into plate count agar (PCA) (Difco™, France) and cultured with pour-plate method. The cultured PCA plate was then incubated at 37 °C in an incubator for 24 h. Feces sample dilution procedure followed the WHO global foodborne Infections Network [15] and bacteria enumeration procedure was performed following FDA bacteriological Analytic Manual 2019 [2].

##### Coliform culture method

One gram of sample was weighed, mixed, and centrifuged until homogenized with PBS and serially diluted 10-fold to obtain 10^− 2^ to 10^− 6^ dilution. One mL of sample suspension was transferred into Violet Red Bile Lactose (VRBL) (Merck™, Germany) and cultured with the pour-plate method. The VRBL plate was cultured at 37 °C in an incubator for 24 h. Further biochemical examinations were tested using Indole, Methyl red, Voges Proskauer, and Citrate media (IMVPC) with interpretation (++--) or (-+--), considered as *E. coli* [16].

##### Escherichia coli culture method

One gram of fecal sample was weighed, mixed, centrifuged and suspended with PBS and serially diluted 10-fold to obtain 10^− 1^ to 10^− 5^ dilution. One mL of sample suspension was transferred into MacConkey agar (OXOID™) and cultured with spread-plate method. The MCA plate was cultured at 37 °C in an incubator for 24 h.

##### Lactic acid producing bacteria culture method

One gram of sample was weighed, mixed, and centrifuged until homogenized with PBS and serially diluted 10-fold to obtain 10^− 3^ to 10^− 7^ dilution. One mL of sample suspension was transferred into deMann Rogosa (MRS) agar (OXOID™) and cultured with the pour-plate method. The MRS plate was cultured at 37 °C in an incubator for 48 h.

For all methods, the overnight culture plates with colony number ranged 25–250 colonies were counted using CFU/gram.

CFU/gram = (sum of colonies counted on the successful plates contains 25–250 colonies X dilution factor of sample) / volume of inoculum applied in a dish

##### Pig growth performance analysis

The parameters used to measure pig performance during the nursery period included the total number of pigs in and out, percent of culled pig, average daily feed intake (ADFI), average daily gain (ADG) and feed conversion ratio (FCR). Weight gain was calculated based on weight at 28 and 56 days. ADG, ADFI and FCR were calculated using this following formula:

ADG (g/d) = weight gained / total day of experiment

ADFI (g/d) = (total feed offered – total feed refused) / total day of experiment

FCR = ADFI / ADG

### Statistical analysis

In experiment 1, sow and piglet performance parameters were analyzed using student *t-test* and paired *t-test*. In experiment 2, growth performance in nursery pigs and the amount of fecal bacterial population (CFU/g) were analyzed using student *t*–test. Significance level was set to *p* < 0.05.

## Results

Under field conditions in a commercial farrow-to-finish farm, the impact of P(OA + EO) supplementation in sows and weaning pigs was carefully evaluated by measuring sow productivity and pig growth performance.

### Experiment 1: Sow experiment

#### Feed analysis

The analyzed proximate and mineral concentrations of the Control and P(OA + EO) group were in alignment with the expected values. The results are shown in Table 1.

**Table 1:** Chemical composition analysis of control and P(OA + EO) feed in sow experiment

Nutrients	Group		Standard**	Source
	Control	*P*(OA + EO)*		
Gross energy (Kcal/kg)	4072	4070	no data	Automatic Bomb Calorimeter; Leco model AC – 500
Moisture (%)	7.83	7.90	no data	
Crude fat (%)	7.20	7.80	no data	
Ash (%)	6.83	6.60	no data	
Phosphorus (%)	0.57	0.60	0.54–0.65	(NRC, 2012)
Calcium (%)	2.01	1.93	0.63–0.76	(NRC, 2012)
Crude protein (%)	15.27	14.96	16.30–19.20	(NRC, 1998)
Crude fiber (%)	5.67	5.31	no data	

*The P(OA + EO) group feed is basal feed topped with 10 g/sow/day of P(OA + EO)

**Based on NRC nutrient requirements of lactating sow (90% dry matter)

Note: 4% lysine was added in the feed based on data obtained from farm owner, nevertheless, % lysine in feed was not determined

#### Effect of supplementation P(OA + EO) on sow performances

The average parity of the sows used in the Control and P(OA + EO) groups was 3.17 ± 1.99 and 2.96 ± 1.93, respectively. With respect to measures related to sow body performance, the average BCS before farrowing and at weaning of the Control (3.09 ± 0.42 and 2.84 ± 0.35, respectively) and of the P(OA + EO) groups (3.15 ± 0.43 and 2.93 ± 0.47, respectively) showed a similar declining trend, which indicate a BCS loss. However, the extent of BCS loss in the P(OA + EO) group was significantly lower than the Control group (0.26 ± 0.25 and 0.23 ± 0.33, respectively, *p* < 0.05). Similarly, the average BF thickness was significantly decreased from late gestation phase (7 days before farrowing) to weaning (28 days after farrowing) in both the Control (17.73 ± 2.42 and 15.03 ± 1.68 mm, respectively) and P(OA + EO) groups (18.48 ± 2.39 and 16.33 ± 2.13 mm, respectively) (*p* < 0.05). Consistent with the results obtained in the BCS loss, the average BF loss of P(OA + EO) group (2.14 ± 0.87 mm) was again significantly lower than the Control group (2.70 ± 1.10 mm) (*p* < 0.05) resulting to a positive correlation between BCS and BF (*p* = 0.01, *r* = 0.362). The percent of sow with shoulder ulcer and the average shoulder score of sows in the P(OA + EO) group was significantly lower (0.55 ± 0.70 and 0.91 ± 1.16, respectively) than the Control group (0.81 ± 0.78 and 1.36 ± 1.30, respectively). Finally, the average feed intake of sows in P(OA + EO) group (5.51 ± 0.12 kg/d) was statistically higher than the Control group (5.40 ± 0.08 kg/d, *p* < 0.05). The results are shown in Table 2.

**Table 2:** Effect of P(OA + EO) on sow performance (data shown as average ± standard deviation)

Sow performance	Group			
	Control (*n* = 167)		P(OA + EO) (*n* = 167)	
	7 days pre-farrowing	28 days post-farrowing	7 days pre-farrowing	28 days post-farrowing
**Sow body performances**				
Average parity of sows tested	3.17 ± 1.99		2.96 ± 1.93	
BCS	3.09 ± 0.42^**b**^	2.84 ± 0.35^**a, x**^	3.15 ± 0.43^**b**^	2.93 ± 0.47^**a, y**^
BCS loss	-0.26 ± 0.25^b^		-0.23 ± 0.33^a^	
BF (mm)	17.71 ± 2.43^**x**^	15.01 ± 1.68^**a, y**^	18.46 ± 2.38^**x**^	16.23 ± 2.46^b, y^
BF loss (mm)	-2.70 ± 1.10^b^		-2.14 ± 0.87^a^	
Shoulder ulcer (%)	0.81 ± 0.78^b^		0.55 ± 0.70^a^	
Shoulder ulcer score	1.36 ± 1.30^b^		0.91 ± 1.16^a^	
ADFI (kg/d)	5.40 ± 0.08^a^		5.51 ± 0.12^b^	
**Sow reproductive performance**				
Total born/litter	13.90 ± 3.05		14.22 ± 3.10	
Born alive /litter	13.49 ± 3.12		13.86 ± 3.35	
Stillbirth (%)	2.32 ± 6.49		2.43 ± 10.55	
Wean-to-service interval (d)	3.91 ± 1.02		4.01 ± 0.87	
**Piglet performances**				
Birth weight (kg)	1.40 ± 0.23		1.39 ± 0.21	
Suckling pig diarrhea (%)	29.15 ± 13.17^b^		24.63 ± 11.73^a^	
Preweaning mortality (%)	15.50 ± 0.99^b^		14.70 ± 1.28^a^	
No. of weaned pig/ litter	11.37 ± 0.49^b^		11.83 ± 0.83^a^	
Piglet weaning weight (kg)	5.90 ± 0.32^b^		6.11 ± 0.34b^a^	
ADLWG (g/d)	1801.40 ± 144.99^b^		1962.80 ± 152.28^a^	

Means in each row with different superscripts were statistically significantly difference (*p* < 0.05)

^a, b^ comparison between control and P(OA + EO) groups

^x, y^ comparison within groups (7 days pre-farrowing and 28 days post-farrowing)

BCS = body condition score

BF = backfat

ADFI = average daily feed intake

ADLWG = average daily litter weight gain

In terms of sow reproductive performance, the average number of total born/litter (14.22 ± 3.10 vs. 13.90 ± 3.05 pigs), average born alive/litter (13.86 ± 3.35 vs. 13.49 ± 3.12 pigs), average of percent of stillbirth (2.43 ± 10.55 vs. 2.32 ± 6.49 pigs), and wean-to-service interval (4.01 ± 0.87 vs. 3.91 ± 1.02 days) were not statistically different between the P(OA = EO) and the Control groups, respectively. (Table 2)

#### Effect of supplementation P(OA + EO) on preweaning pig performances

The average birth weight of piglets in P(OA + EO) group (1.39 ± 0.21 kg) was not statistically different from that of the Control group (1.40 ± 0.23 kg). The number of pigs with diarrhea per litter (3.18 ± 1.02 vs. 3.86 ± 1.11 pigs), percentage of pigs with diarrhea (24.63 ± 11.73 vs. 29.15 ± 13.17%) and percent of preweaning mortality (14.70 ± 1.28 vs. 15.50 ± 0.99%) were significantly lower in the P(OA + EO) group than in the Control group. On the other hand, the average number of weaned pigs per litter (11.83 ± 0.83 vs. 11.37 ± 0.49 pigs), average pig weaned weight/litter (6.11 ± 0.34 vs. 5.90 ± 0.32 kg), and the average daily litter weight gain (1962.80 ± 152.28 vs. 1801.40 ± 144.99 g/d) were statistically higher in the P(OA + EO) than in Control group. (Table 2)

### Experiment 2: nursery pig experiment

#### Feed analysis

The analyzed proximate and mineral concentrations of the Control and the P(OA + EO) during the Phase I and II of the nursery periods were in alignment with the expected values. However, percent calcium P(OA + EO) phase II diet (2.0%) was moderately higher than the Control diet (1.4%). The results are shown in Table 3.

**Table 3:** Chemical composition analysis of control and treatment feed in nursery pig experiment

Nutrients	Group			Standard**	Source
	Control	*P*(OA + EO)			
		N1	N2		
Gross energy (Kcal/kg)	4099	4124	4130	no data	Automatic Bomb Calorimeter, Leco model AC – 500
Moisture (%)	8.65	8.71	8.24	no data	
Crude fat (%)	7.05	7.27	7.41	no data	
Ash (%)	6.14	5.77	6.21	no data	
Phosphorus (%)	0.62	0.62	0.62	0.60–0.70	(NRC, 2012)
Calcium (%)	1.40	1.63	2.00	0.70–0.85	(NRC, 2012)
Crude protein (%)	18.38	18.59	17.8	18.0–23.7	(NRC, 1998)
Crude fiber (%)	2.90	3.35	3.18	no data	

*Based on NRC (2012) of nursery pigs with body weight ranged 5–25 kg when feed allowed old piglets *ad libitum* (90% dry matter)

**The results of gross energy were determined by Automatic Bomb Calorimeter; Leco model AC–500

**N1** treatment = P(OA + EO) 2 kg/ton of basal feed, control = basal feed, 28–49 days old piglets

**N2** treatment = P(OA + EO) 1 kg/ton of basal feed, control = basal feed, 50–70 days old piglets

Note: 4% lysine was added in the feed based on data obtained farm owner, nevertheless, % lysine in feed was not determined

#### Effect of supplementation P(OA + EO) on nursery pig growth performances

Four sets of nursery pig experiment were conducted in parallel with sow experiment. No relationship between sow and nursery experiment was declared. The ADFI (602.04 ± 39.39 vs. 582.43 ± 46.41 g/d) and ADG (437.48 ± 30.70 vs. 402.27 ± 24.67 g/d) of pigs in the P(OA + EO) group were significantly higher (*p* < 0.05) than the Control group. On the other hand, the FCR in the P(OA + EO) group was significantly lower (1.37 ± 0.07) than the Control group (1.44 ± 0.06). There was no significant difference in other growth performance parameters considered during the nursery period). The details are shown in Table 4.

**Table 4:** Effect of P(OA + EO) on nursery pig performance (data obtained from 4 replicates for a total of 3065 pigs and shown as average ± standard deviation)

Parameters	Group	
	Control	*P*(OA + EO)
No. of nursery pig-in	1539	1526
No. of nursery pig-out	1521	1493
Average nursery pig weight in (kg/pig)	5.71 ± 0.08	5.57 ± 0.37
Average nursery pig weight out (kg/pig)	22.35 ± 2.56	23.52 ± 2.25
ADFI (g/d)	582.43 ± 46.4^b^	602.04 ± 39.39^a^
ADG (g)	402.27 ± 24.67^b^	437.48 ± 30.70^a^
FCR	1.45 ± 0.06^b^	1.38 ± 0.07^a^
Average nursery pig mortality (%)	1.15 ± 0.42	2.12 ± 0.97

^a, b,^ means in each row with different superscripts were statistically significantly difference (p < *0.05)*

ADFI = average daily feed intake

ADG =average daily gain

FCR = feed conversion ratio

#### Effect of supplementation P(OA + EO) on fecal bacteria population

The results of P(OA + EO) supplementation in nursery pig feed on selected fecal bacterial population are shown in Table 5. At the beginning of the experiment (day 28), the average total bacteria number in the P(OA + EO) (8.03 ± 0.65 CFU/g) was significantly lower than the Control group (8.37 ± 0.54 CFU/g) (*p* < 0.05). However, the average numbers of coliform bacteria, *E. coli*, and Lactobacillus spp. were not statistically different between P(OA + EO) and the Control groups.

**Table 5:** Effect of P(OA + EO) supplementation in feed on fecal bacterial population in nursery pig experiment

Fecal Bacterial population (log10 CFU/g)	Groups					
	28 days of age		49 days of age		70 days of age	
	Control*	*P*(OA + EO)	Control*	*P*(OA + EO)	Control*	*P*(OA + EO)
Total bacteria	8.37 ± 0.54^b^	8.03 ± 0.65^a^	7.04 ± 0.80	7.10 ± 0.77	6.43 ± 1.42^a^	7.27 ± 0.57^b^
Coliform bacteria	4.88 ± 2.09	4.65 ± 1.79	4.33 ± 1.42	4.46 ± 1.33	3.79 ± 2.48^a^	5.68 ± 1.46^b^
*E. coli*	3.97 ± 2.66	3.47 ± 2.50	2.72 ± 2.14	3.42 ± 1.72	3.22 ± 2.41^a^	4.95 ± 1.79^b^
Lactobacillus spp.	7.81 ± 0.74	7.56 ± 0.59	5.90 ± 1.29	6.25 ± 0.89	5.44 ± 1.24^b^	6.33 ± 0.75^a^
*L/T ratio*	0.93 ± 0.06	0.94 ± 0.07	0.86 ± 0.07	0.88 ± 0.08	0.86 ± 0.21	0.87 ± 0.07
*L/C ratio*	1.77 ± 0.89	2.06 ± 1.34	1.37 ± 0.47	1.39 ± 0.54	1.08 ± 0.90	1.21 ± 0.44

*Control (basal feed without supplementation) from 28–70 days of age, P(OA + EO) feed I [basal feed + P(OA + EO) 2 kg/ton of feed] at 28–49 days of age, and P(OA + EO) feed II [basal feed + P(OA + EO) 1 kg/ton of feed] at 50–70 days of age

L/T *=* Lactobacillus spp./Total bacteria ratio. L/C *=* Lactobacillus spp./Coliform bacteria ratio

^a, b,^ means in each row with different superscripts were statistically significantly difference (*p* < 0.05)

At middle of experiment (day 49), all bacterial population parameters were not statistically different in both treatments. Interestingly, there appeared to be a higher number in P(OA + EO) than the Control group.

At the end of experiment (day 70), the total bacteria number in the P(OA + EO) (7.27 ± 0.57 CFU/g) was significantly higher than the Control group (6.43 ± 1.42 CFU/g). Similarly, the average numbers of coliform bacteria (5.68 ± 1.46, vs. 3.79 ± 2.48), *E. coli* (4.95 ± 1.79 vs. 3.22 ± 2.41), and Lactobacillus spp. (6.33 ± 0.75 vs. 5.44 ± 1.24 CFU/g) in the P(OA + EO) group were significantly greater (*p* < 0.05) than the Control group respectively. Additional investigation on the ratio of L/T and L/C did not show statistical difference between two groups.

## Discussion

The effects of microencapsulated blend of organic acids and essential oils on sow productivity and nursery pig performance were investigated under commercial field conditions. The study outcome in the sow experiment revealed a better BCS score and BF thickness in the P(OA + EO)-supplemented group than in the Control-non-supplemented group. Accordingly, BCS and BF losses from farrowing to weaning were also better in the P(OA + EO) group than in the Control group. The examination of shoulder ulcer was supportive of this finding as incidence and severity of shoulder ulcer were lower in the P(OA + EO) supplemented sows compared with the non-supplemented sows. The reduction in backfat thickness and BCS are commonly observed in multiparous sow, attributable to the challenges associated with multiple farrowing events [17]. A previous study [18] indicated that reduction of body weight and BF thickness may be decreased during lactation, if the sows were dietary supplemented with 0.8% potassium diformate during pre-farrowing to weaning period [9]. Lower feed intake during the lactating period directly causes loss of BF thickness and poor BCS due to mobilization of body fat and protein reserves [19]. An earlier report suggested that pregnant sow had elevated oxidative stress during gestation and lactation periods. This could lead to excessive production of reactive oxygen species (ROS) in the blood, which causes insulin signaling cascade that leads to insulin resistance [20]. The primary role of insulin is to control glucose homeostasis by stimulating glucose transport into muscle and adipose cells, while reducing hepatic glucose production via gluconeogenesis and glycogenolysis. Insulin also regulates lipid metabolism by increasing lipid synthesis in liver and fat cells while inhibiting lipolysis [21]. Insulin resistance can have a negative effect on sow feed intake during lactation [22] and coupled by the oxidative stress experienced during this period, lipolysis can occur, causing reduction in body weight and BF thickness during lactation. Tan et al. [23] reported that adding 15 mg/kg feed of oregano essential oil (carvacrol and thymol) into multiparous sow diet could increase feed intake in the third week of lactation period. This report supports the result in this study, suggesting that supplementing essential oil in lactating sows improves feed intake causing lower BCS and BF losses at weaning. However, blood profile was not determined in this study to confirm the effect of P(OA + EO) addition in late gestation to lactation in terms of mitigating oxidative stress and its corresponding effect on insulin resistance.

In this study, P(OA + EO) supplementation in sow feed had no significant effect on piglet born alive. This is because the supplementation period in this study was one week before farrowing and therefore, the process of fertilization which can impact this parameter has already taken place. To achieve a better born alive number of piglets, it requires to have an appropriate timing and technique of insemination and good management during the first month of gestation. Further study with a different feeding program of P(OA + EO) is necessary to determine if it can positively affect the number of piglets born alive. Similar finding was observed by Balasubramanian et al. [9], who reported that the administration of organic acid blends did not show significant results in increasing the number of live piglets. In this study, stillbirth numbers of both groups were low and not affected by treatment. Weaning weight was increased by P(OA + EO) supplementation and was associated in part with greater milk production as evidenced by the higher litter growth in sows supplemented with P(OA + EO).

In this study, the increased number of weaning pigs from sow supplemented with P(OA + EO) may be associated with increased intake of sow colostrum which can provide passive immunity and energy for growth and thermoregulations for weaned piglets during their first 24 h [13, 24]. Ariza- Ariza-Nieto et al. [25] reported that adding essential oils such as thymol could increase the T-lymphocyte number in the blood and milk, demonstrating the efficacy of thymol as an immunostimulant for the suckling pig which can improve their survival until weaning phase. Supplementing 0.2% protected organic acid could improve white blood cell counts in suckling pigs and therefore, the combination of these molecules may result to better production outcomes [26].

There was no difference observed in the weaning to service interval of sows between the 2 treatment groups. This is in accordance with a previous report [9]. However, there is also associated evidence that weaning to service interval is influenced by the extent of body weight loss during lactation [27]. If feed intake during lactation is maximized, the probability of sow body weight loss from late gestation to weaning is reduced, resulting to shorter weaning to service interval [28].

In this study the supplementation of P(OA + EO) in nursery diet improved all aspects of piglet growth performance. Yan et al. [29] reported that a mixture of essential oils and organic acids consisting of cinnamaldehyde (15%), thymol (5%), citric acid (10%), sorbic acid (10%), malic acid (6.5%) and fumaric acid (13.5%) increased ADG of 21–49 days old, crossbred piglets (Duroc × Landrace × Yorkshire). In addition, Upadhaya et al. [30] also reported that 0.2% protected organic acids (MCFA and composite organic acids) were able to improve performance and ADG in growing pigs. Results related to the effect of giving essential oils and organic acids to improve the performance of weaning pigs were also reported [31, 32]. Nonetheless, Lee et al. [33] and Manzanilla et al. [34] report that the administration of a single acidifier or blend of acidifiers did not have a positive effect on the growth performance of pigs. This inconsistency may be due to the beneficial effect of microencapsulation which protects the organic acids from dissociation as they pass through the different sections of the GI tract, particularly in the hindgut where they are susceptible to dissociation. Organic acids in their undissociated form are more effective in inhibiting pathogenic bacteria that mostly thrive in the hindgut. Previous studies showed that organic acid and essential oil blends improved pig diarrhea [35] and pig performance although the microencapsulation may have provided higher efficiency at lower dosages [8].

The FCR of P(OA + EO) supplemented nursery piglets was also improved during the phase I period (28–49 days old). Yan et al. [29] revealed that supplementation of essential oils (thyme, rosemary, and oregano extracts) resulted to a better FCR in nursery pigs. Another report [36] demonstrated that application of a mixture of formic, propionic and caprylic acid in the feed of pigs aged 35–56 days significantly increased feed efficiency. This result can be again attributed to the effect of microencapsulating organic acids and essential oils, helping them be more effective in the lower part of the GI tract. On the contrary, the FCR of pig during the phase II period (50–70 days old) did not differ between the 2 treatment groups. The inconsistency in effects may be associated with the dose and duration of the acidifier supplementation [37].

The effect on fecal bacterial population following dietary supplementation of P(OA + EO) showed significant differences in total bacterial counts at the beginning (28 days old) and at the end (70 days old), but not at the middle of the experiment (49 days old). The high bacterial counts at the beginning of the weaning phase may be due to many stress factors such as the separation of the piglets from the sow, the shift in feeding pattern, and the abrupt change in piglet nutrition. Such periods of stress can result in an imbalance of the gastrointestinal microbiota, which allows opportunistic pathogens to proliferate and cause GI-tract disturbances [38]. A similar opinion was shared by Partanen and Mroz [5] that in the early post-weaning phase, pigs often have an overgrowth of pathogenic bacteria (e.g., coliforms) due to the high gastric pH resulting from an increase in the amount of undigested feed entering the GIT. In this study, we found markedly increased numbers of coliform bacteria, *E. coli*, and Lactobacillus spp. in the feces of P(OA + EO)-supplemented pigs, compared to the Control non-supplemented pigs, on day 70. In contrast, no significant difference in fecal bacterial counts were found on days 28 and 49 in both treatment groups. The implication of this observation may indicate the need for an adequate period of P(OA + EO) supplementation to amend the bacterial population in pig feces.

The increased number of Lactobacillus species. in this study is similar with the study of Lan and Kim [39], which examined supplementation of 0.2% of OA blend (fumaric, citric, malic, capric, caprylic acids, and kaolin) in suckling piglets and showed an increased amount of Lactobacillus species and reduced amount of *E. coli.* Zeng et al. [32] similarly reported that adding a combination of essential oil (0.025% cinnamaldehyde and thymol) slightly increased the number of Lactobacillus species and reduced *E. coli* number in feces of weaning pig. By applying a microencapsulation technology, the targeted release of active compounds like OA and EO in closed proximity of bacteria will cause damage to the cell walls of coliform bacteria which are sensitive to acid, which in turn inhibits the process of proliferation of bacteria that are sensitive to acid [40]. Knarreborg et al. [41] explained in a previous report that in vitro simulation of the growth of lactic acid bacteria vs. coliform to mimic major environments of stomach and proximal part of piglet small intestines displayed that a population of coliform cannot grow in stomach contents. The Lactobacillus population and L: C ratio of P(OA + EO) supplemented pigs in this study revealed a trend towards a higher number and ratio than the Control non-supplemented pigs. This suggests that P(OA + EO) supplementation provides gut environment conditions to be suitable for beneficial bacteria to be grown and offers a better health and growth performance of pigs.

## Conclusion

The outcomes of this study provide compelling evidence that the inclusion of P(OA + EO) in lactating sow and nursery diets contributes to increased sow productivity during lactation, enhanced performance of suckling and nursery piglets. These findings suggest the potential for P(OA + EO) as a valuable nutritional supplement in swine management practices, offering a holistic approach to optimizing swine health and productivity. Further research is warranted to better elucidate the underlying mechanisms regarding its effect on gut microbial profile particularly during the critical period of weaning.

## Data Availability

Information involving the product of this study is available in previous publications: doi: 10.1093/tas/txz176, doi: 10.1093/jas/skaa259, and doi: 10.1016/j.aninu.2020.04.004.
